# A Study on Measurement Variations in Resonant Characteristics of Electrostatically Actuated MEMS Resonators

**DOI:** 10.3390/mi9040173

**Published:** 2018-04-09

**Authors:** Faisal Iqbal, Byeungleul Lee

**Affiliations:** 1 Interdisciplinary Program in Creative Engineering, Korea University of Technology and Education, Cheonan 31253, Republic of Korea; faisal@koreatech.ac.kr; 2 School of Mechatronics Engineering, Korea University of Technology and Education, Cheonan 31253, Republic of Korea

**Keywords:** electrostatic actuation, MEMS resonator, measurement variation, frequency domain, resonant characteristics

## Abstract

Microelectromechanical systems (MEMS) resonators require fast, accurate, and cost-effective testing for mass production. Among the different test methods, frequency domain analysis is one of the easiest and fastest. This paper presents the measurement uncertainties in electrostatically actuated MEMS resonators, using frequency domain analysis. The influence of the applied driving force was studied to evaluate the measurement variations in resonant characteristics, such as the natural frequency and the quality factor of the resonator. To quantify the measurement results, measurement system analysis (MSA) was performed using the analysis of variance (ANOVA) method. The results demonstrate that the resonant frequency ($f_{r}$) is mostly affected by systematic error. However, the quality (Q) factor strongly depends on the applied driving force. To reduce the measurement variations in Q factor, experiments were carried out to study the influence of DC and/or AC driving voltages on the resonator. The results reveal that measurement uncertainties in the quality factor were high for a small electrostatic force.

## 1. Introduction

Microelectromechanical systems (MEMS) resonators are the basic building blocks for most MEMS devices, such as gyroscopes and accelerometers [1,2,3,4]. A variety of research has been carried out to improve MEMS structure designs [5,6,7] and fabrication processes [8,9,10]. Testing of MEMS devices is one of the important aspects. The test system needs to be cheap, and must have the capability to produce fast and accurate results [11,12].

In most MEMS devices, resonant characteristics, such as resonant frequency and the quality (Q) factor, are of particular interest. These characteristics help designers revise the MEMS structure layout, for better performance and productivity. The resonant characteristics can be measured by either optical [8,13] or electrical methods [14]. Optical testing has the advantage of high accuracy, but is more complicated and expensive, compared to electrical testing.

One of the problems in measuring MEMS resonant characteristics is measurement variations. Research efforts have been made to find the reasons for these variations. They may be caused by temperature [15] or pressure [16], as well as the actuation scheme [17]. Lee et al. made efforts to measure the uncertainty in resonant characteristics based on different actuation schemes [18]. Characterization of the devices was obtained using an optical method. The results demonstrated that electrostatic actuation exhibits higher random uncertainties in quality factor measurement, compared to piezo-electric actuation. However, resonant frequency measurement is independent of the actuation scheme.

MEMS resonators can be actuated using different techniques, such as piezoelectric, thermal [19], and electrostatic [18]. Among different actuation schemes, electrostatic actuation is preferred due to fast response, ease of fabrication, and compatibility with the CMOS process.

For electrostatic actuation, electrostatic force is generated by exploiting the parallel plate capacitor. Two approaches can be used for electrostatic actuation, i.e., varying gap and varying area. An AC superimposed on DC voltage is applied to one side of the parallel plate, while the other side is grounded. This will vibrate the structure in response to the drive frequency of the AC. In the varying gap method, also known as parallel plate actuation, the electrostatic force is a nonlinear function of displacement [14,20], which results in an electrostatic, negative spring effect. The effect of nonlinearity and the negative spring effect have been studied in [21,22,23,24,25,26,27]. On the other hand, the varying area method, also known as comb drive actuation, does not result in negative spring constant, when the fringe field is negligible [28].

For electrostatic actuation and capacitive sensing, an electrical method is commonly used to measure the resonant characteristics. There are two approaches to measuring resonant characteristics electrically. One is the time domain method, and the other is the frequency domain method. In the time domain method, the time decay signal of the resonator is recorded, and the quality factor is evaluated [29,30]. In the frequency domain, the frequency is swept around the resonant frequency, which produces a complex frequency response function (FRF). The resonant characteristics are computed from the FRF. Frequency domain analysis is a simple and fast method [14,31,32,33]. To the best of the authors’ knowledge, measurement uncertainties in the resonant characteristics using frequency domain analysis without a fitting method have not been investigated thoroughly.

In this paper, we measure resonant characteristics of the resonator directly from the signal analyzer. This method is simple, fast, and cost-effective, when mass-fabricated devices need to be tested. We try to minimize and quantify the measurement errors in a capacitive MEMS resonator, using frequency domain analysis. First, measurement system analysis (MSA) was performed with a conventional method (referred to as condition 1), to study the measurement uncertainties. The analysis of variance (ANOVA) method, which is based on repeatability, reproducibility, and part-to-operator interaction, was used. To quantify the measurement variations, additional experiments were carried out, to study the effect of applied alternating current (AC) and/or direct current (DC) voltages on the measurement variations. We showed that optimal values (referred as condition 2) for the AC and DC voltages exist, which result in smaller measurement variations. The optimal driving conditions were further verified by the MSA test.

## 2. Experimental Setup

**Figure 1:**
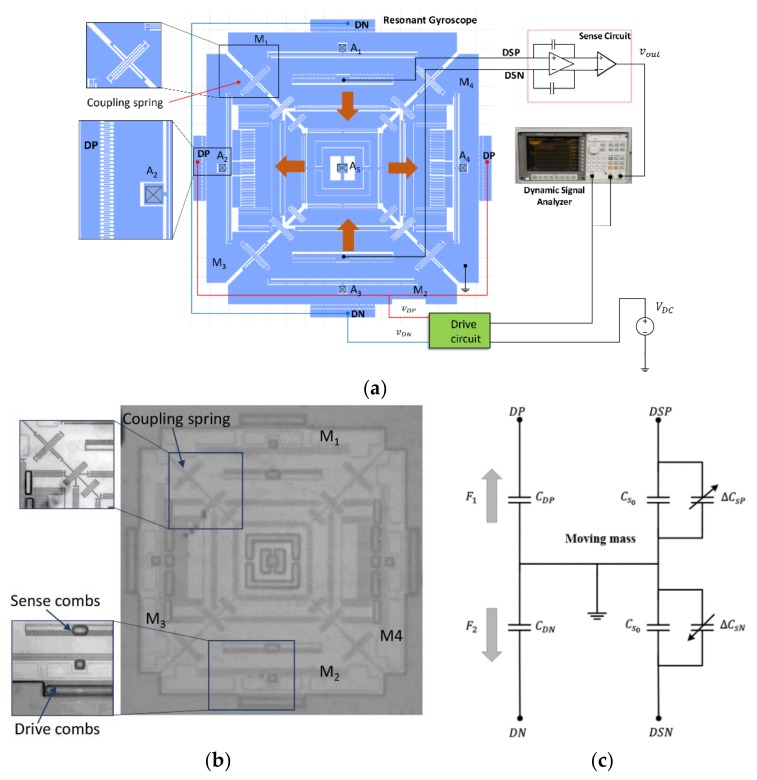
(**a**) Experimental setup for resonant characteristics measurement of the MEMS resonator. (**b**) Optical photograph of the vacuum packaged single structure 3-axis gyroscope. (**c**) Electrical model of the MEMS resonator.

This study was conducted using a vacuum-packaged, single-structure, three-axis gyroscope presented by Shah et al. [34]. The structure comprises four driving masses, coupled through coupling springs. Where two masses, $M_{1}$ and $M_{2}$, move inwards, masses $M_{3}$ and $M_{4}$ will move outward. The masses were fixed at five anchor positions, named $A_{1}$–$A_{5}$, as shown in Figure 1a. Such a configuration was used to reduce the effect of slide film damping, and improve the Q factor of the drive mode. The four masses were actuated differentially, and differential sensing was used to reject the linear acceleration. 

In this paper, we choose to investigate the resonant characteristics of the drive mode only. The drive mode dynamics are given as:

(1) $$F_{d}=M_{d}\frac{d^{2}x}{dt^{2}}+D_{d}\frac{dx}{dt}+K_{d}x$$

where $M_{d},D_{d},K_{d}$ means the mass, damping, and stiffness of the drive mode of the gyroscope, respectively. $F_{d}$ is the applied external driving force. The transfer function from the drive force, $F_{d}$, to the drive-mode vibration displacement, x, can be written as:

(2) $$H\left(s\right)=\frac{X\left(s\right)}{F\left(s\right)}=\frac{\frac{1}{M_{d}}}{s^{2}+2\zeta \omega_{r}s+\omega_{r}^{2}}$$

where $\omega_{r}=\sqrt{K_{d}/M_{d}}$ and $\zeta =D_{d}/2M_{d}\omega_{r}$ are the resonant frequency and damping factor of the drive mode resonator. The displacement magnitude and phase response as a function of frequency are given by:

(3) $$X\left(\omega \right)=\frac{F_{d}/K_{d}}{\sqrt{{\left[1-\frac{\omega^{2}}{\omega_{r}^{2}}\right]}^{2}+{\left[2\zeta \frac{\omega }{\omega_{r}}\right]}^{2}}}$$

and:

(4) $$\varphi \left(\omega \right)=\tan^{-1}\frac{2\zeta \frac{\omega }{\omega_{r}}}{1-{\left(\frac{\omega }{\omega_{r}}\right)}^{2}}$$

Device fabrication and gyroscope evaluation are beyond the scope of this paper. Figure 1a represents the measurement setup used for the experiment. Figure 1b represents the fabricated device, and Figure 1c shows the electrical equivalent model of the resonator.

The device was actuated laterally using comb drive. A balance actuation scheme was used to mitigate the effect of nonlinear force with respect to DC voltage and AC voltage. The net electrostatic force experienced by the resonator is directly proportional to the applied voltage, given as:

(5) $$F_{1}=\frac{1}{2}\frac{dC_{DP}}{dx}V_{DP}^{2}$$

(6) $$F_{1}=\frac{1}{2}\frac{dC_{DN}}{dx}V_{DN}^{2}$$

(7) $$F_{d}=F_{1}-F_{2}=\frac{dC_{D}}{dx}\;V_{DC}V_{AC}$$

where $C_{D}$ is driving capacitance, and $V_{DC}$ and $V_{AC}$ are the applied DC and AC voltage, respectively.

A fast sine sweep AC signal, along with an external DC signal, was fed to the drive circuit. The differential drive signals, i.e., $V_{DP}=V_{DC}+V_{AC}$ and $V_{DN}=V_{DC}-V_{AC}$, produced by the drive circuit were applied to the driving electrodes, which were called drive positive (DP) and drive negative (DN). The sense current from the sensing electrodes, drive sense positive (DSP) and drive sense negative (DSP), were converted to voltages through a charge amplifier. After the difference amplifier, the sense voltage was applied to the signal analyzer (Keysight 35670A, Keysight, Santa Rosa, CA, USA) to find the frequency response. The conditions used for the signal analyzer through the experiment are summarized in Table 1.

**Figure 2:**
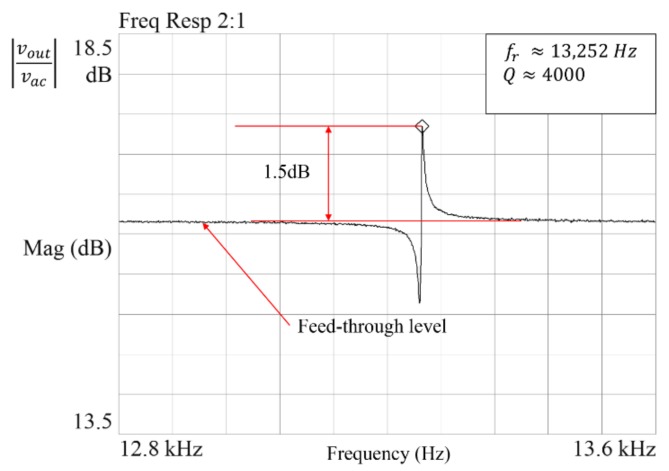
Typical frequency response of a MEMS resonator.

The frequency response function was extracted using frequency domain analysis. A uniform window was selected to measure the results more precisely. A periodic chirp signal centered at the resonance frequency, with a frequency span of 800 Hz centered at the resonant frequency, was used to reduce the test time during the experiment [12]. The typical frequency response of the resonator is shown in Figure 2. The feed-through signal is due to coupling of the drive signal to the sensing signal.

The signal analyzer interpolates the data by itself, and computes the resonant characteristics from FRF. This method is commonly used in industries, for fast computation of the resonant characteristics at a low cost, where-mass fabricated devices need to be tested. The marker function calculates the frequency and damping, using a single degree of freedom curve fitter on the data between start and stop markers. The algorithm computes a conjugate pole pair, implicitly assuming an underdamped system. The resonant frequency $\left(f_{r}\right)$ and damping are computed as follows:

(8) $$Resonant\;frequency\;\left(f_{r}\right)=I_{m}$$

(9) $$Damping\;\left(\zeta \right)=\frac{-R_{e}}{\sqrt{R_{e}^{2}+I_{m}^{2}}}$$

where $R_{e}$ and $I_{m}$ are the real and complex parts of the pole pair corresponding to the resonance. The algorithm used by the signal analyzer works well for high Q resonators, however it will fail to compute the damping for critically- or over-damped systems, since the filter always assumes an underdamped system [35].

## 3. Results and Discussions

### 3.1. Gage R&R for Conventional Method

To study the effect on measurement uncertainties, gage repeatability and reproducibility (Gage R&R) were carried out using condition 1. Table 2 summarizes electrostatic force and Gage R&R conditions. The Gage R&R study was performed using 10 samples with two operators and three trials each. Minitab software (Minitab Inc., State College, PA, USA) was used for ANOVA.

The Minitab statistical results for condition 1 are summarized in Table 3 and Table 4. For the two-way ANOVA part*operator, the *p* value was greater than 0.05; thus, Minitab will automatically calculate the Gage R&R results without interaction. The higher *p* value arises from part-to-part variations.

The percentage contribution of the variance component is zero for the resonant frequency, and 9.6 for the quality factor. Although all the devices have the same design and were fabricated through the same process, the devices have slightly different resonant characteristics, due to fabrication imperfections. For the resonant frequency, all the variations in measurement were caused by parts, while the quality factor measurement suffered from repeatability variations as well.

The Gage R&R percentage study variation for the resonant frequency was 0.4, having 329.0 distinct categories, whereas the Gage R&R percentage study variation for the quality factor was 30.9. The number of distinct categories is only 4.0. High variations of 30.9 were observed for repeatability.

**Figure 3:**
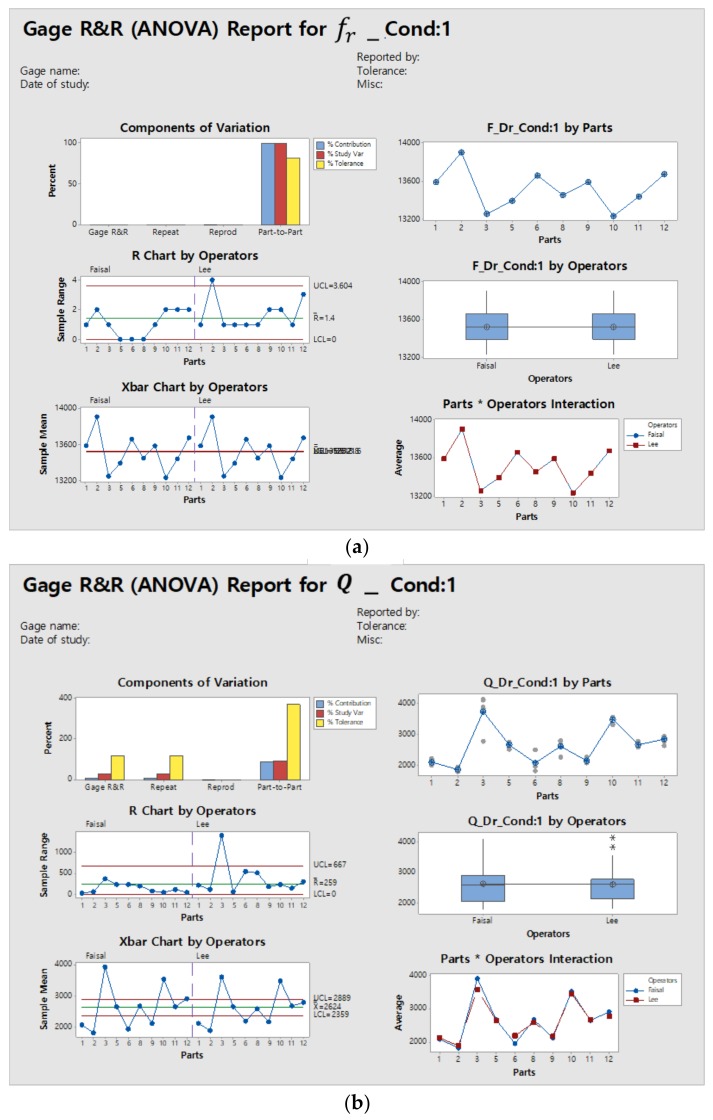
Gage repeatability and reproducibility (R&R) of (**a**) the resonant frequency $\left(f_{r}\right)$ and (**b**) the quality (Q) factor for condition 1.

Since there are no standards for MEMS testing of Gage R&R, we used the Automotive Industry Action Group (AIAG) standards. According to AIAG standards, the measurement results are acceptable if the percentage contribution is less than 1%, and percentage study variations are less than 30% [36]. The results demonstrate that the resonant frequency measurements qualify according to the AIAG standards, however the Q factor does not satisfy the standards. Figure 3a,b illustrate the ANOVA results for both resonant frequency and quality factor, respectively.

The component of variation (subplot top left) in Figure 3a,b gives the assessment of the measurement system. For the resonant frequency ($f_{r}),$ it was observed that the variations are mainly because of the part-to-part, however the quality factor Q is susceptible to measurement variations and failed to reproduce the same results. The R chart (left middle) and Xbar chart (left bottom) show the operator consistency and part-to-part variations due to the repeatability component. The part-to-operator subplot (bottom right) shows the average measurements by each operator for each part. It was observed that the resonant frequency ($f_{r}$) measurement by each operator was almost the same, but there was a lot of variation in the quality factor measurement.

### 3.2. Effect of Driving Voltage on Measurement Variations

To quantify the random uncertainties in Q factor measurement, further experiments were performed. Initially, the AC voltage was kept constant at minimum voltage (200 mV_pk_), and the DC was increased slowly to the maximum possible value. Further increases in the DC voltage resulted in static pull-in, and the FRF becomes unstable. Then, the DC voltage was kept constant (5 V, which is the maximum possible value in our case), and the AC was increased to the maximal value. Further increasing the AC resulted in dynamic pull-in, and the FRF become unstable. The effects of AC voltage and DC voltage were studied independently. Two cases were considered for the rest of the experiment: (i) increasing AC voltage with a fixed DC, and (ii) increasing DC voltage while AC is kept constant.

#### 3.2.1. Increasing AC with a Fixed DC

**Figure 4:**
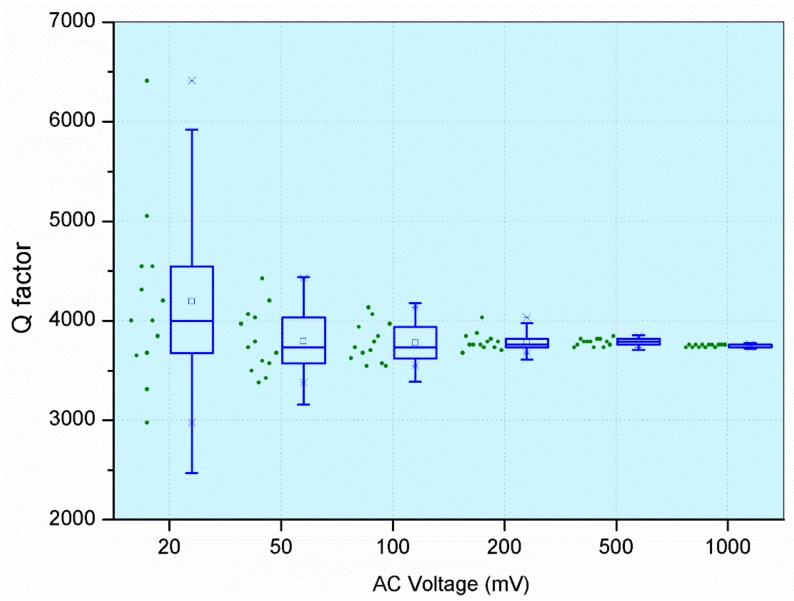
Measurement variations in the Q factor from increasing the AC voltage.

Figure 4 shows the measurement variations when a fixed DC voltage of 5 V was applied to the resonator, and AC was increased from 20 mV_pk_ to 20 V_pk_. A single device was tested 13 times while keeping the test conditions constant. We observed that the random variations were reduced significantly by increasing the AC voltage. Further increases in the AC voltage resulted in instability of the MEMS resonator, due to dynamic pull-in phenomena [20].

**Figure 5:**
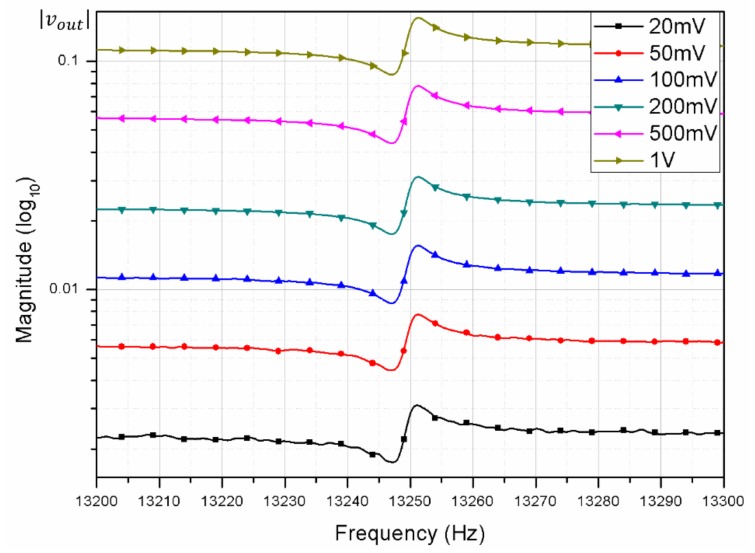
Frequency response of a resonator from increasing the AC voltage at a fixed DC voltage of 1 V.

Figure 5 shows the measured FRF at different AC values. Since electrostatic actuation has the problem of parasitic feed-through, where the drive signal is directly coupled to the sense signal [37,38], we observed that increasing the AC voltage results in much higher force, but the transmission magnitude remains almost constant, due to parasitic feed-through. We also observed that by increasing the AC signal, the effect of random noise in the measured FRF is reduced by high signal amplitude.

#### 3.2.2. Increasing DC with a Fixed AC

**Figure 6:**
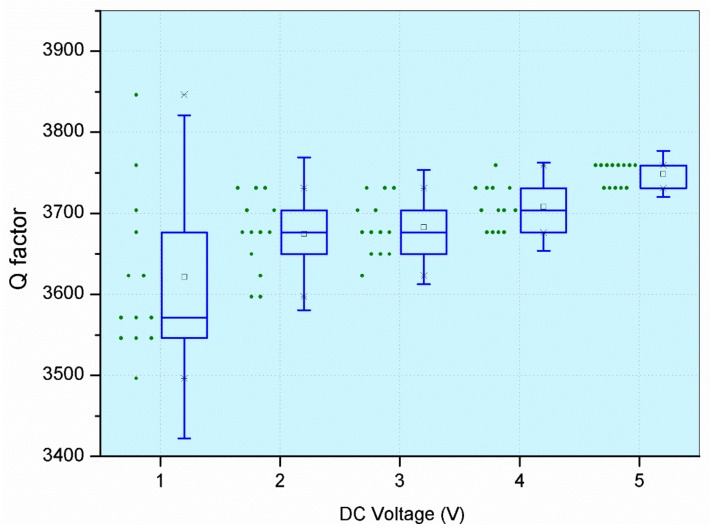
Measurement variations in the Q factor from increasing the DC voltage.

**Figure 7:**
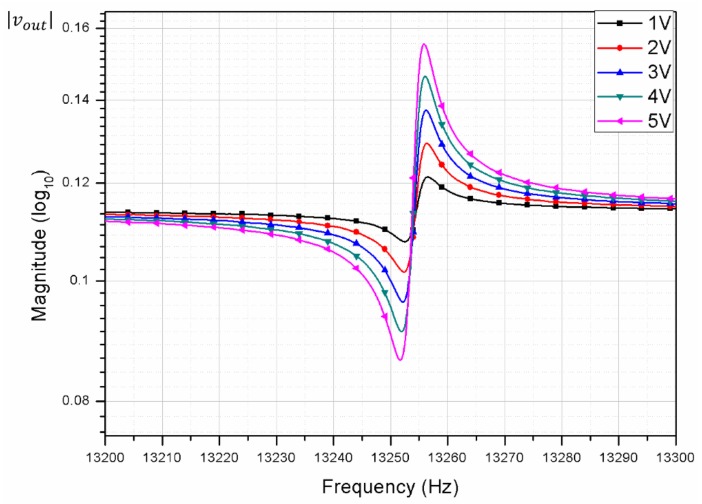
Frequency response of a resonator from increasing the DC voltage at a fixed AC voltage of 1 V_pk_.

Figure 6 shows the measurement variation from increasing the DC voltage and fixing the AC voltage at 1 V_pk_. The same procedure was followed: each device was tested 13 times by a single operator, while keeping the test conditions the same. We observed that by increasing the DC voltage, the measured variations were also reduced. The measured FRF is shown in Figure 7. Random noise in the measured signal was suppressed by high signal amplitude. Also, we observed that by increasing the DC voltage, the transmission magnitude also increased, while the feed-through noise, which depends on the AC voltage only, remained the same.

### 3.3. Gage R&R for Condition 2

Table 5 and Table 6 summarize the Minitab statistical results for condition 2. The percentage contribution of the variance component for the resonant frequency is zero. The Gage R&R percentage study variation is 0.1%, and the results improved by 75%. In addition, the number of distinct categories of the resonant frequency increased to 1184.0.

**Figure 8:**
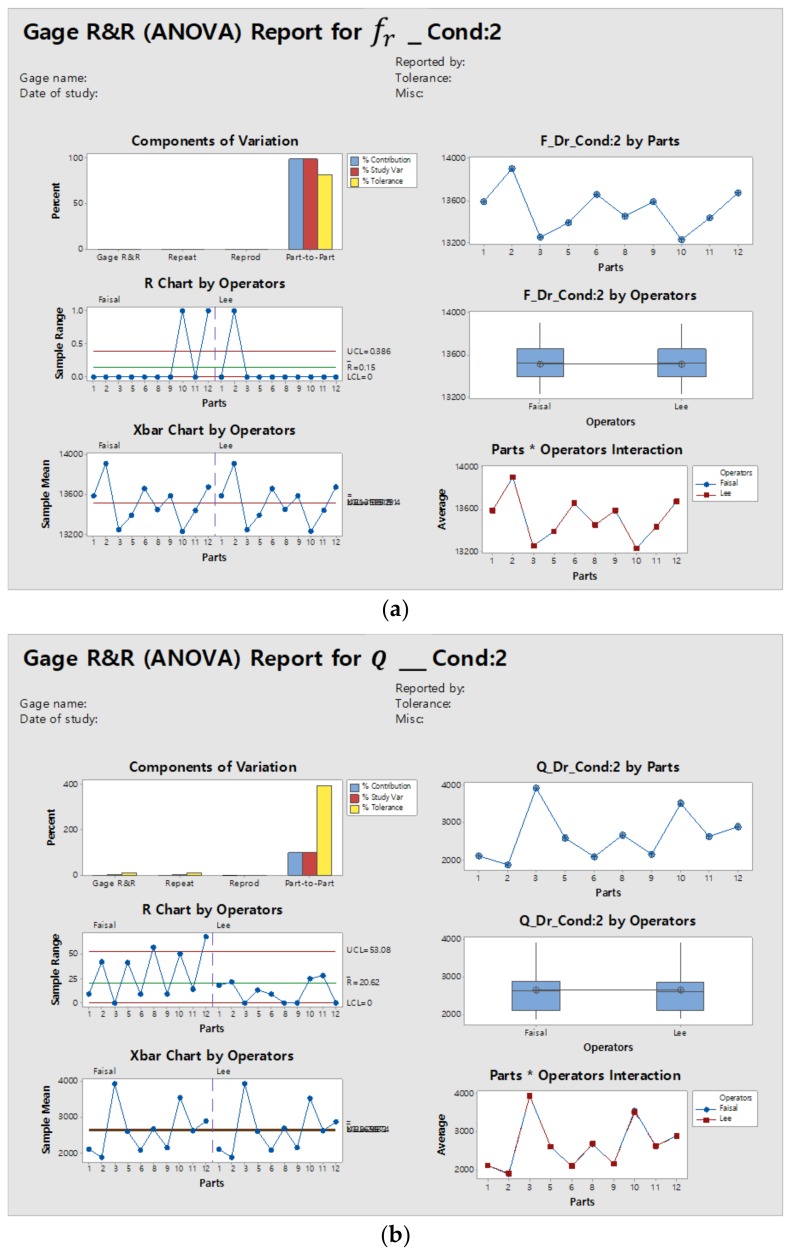
Gage R&R ANOVA results for (**a**) the resonant frequency $\left(f_{r}\right)\;$ and (**b**) the Q factor for condition 2.

For the quality factor measurement in condition 2, we observed that the Gage R&R percentage contribution and study variance were reduced to 0.1% and 2.5%, respectively. The number of distinct categories was also improved to 55, which is a greater than 10-fold improvement. The repeatability variations were reduced to 2.5%. Figure 8a,b show the ANOVA results for the resonant frequency and *Q* factor measurement, respectively.

It was observed that the component of variation for the Q factor has been improved, compared to condition 1. Moreover, the part-to-operator interaction was improved, which means that, for the same device, different operators will read the data with less variation.

**Figure 9:**
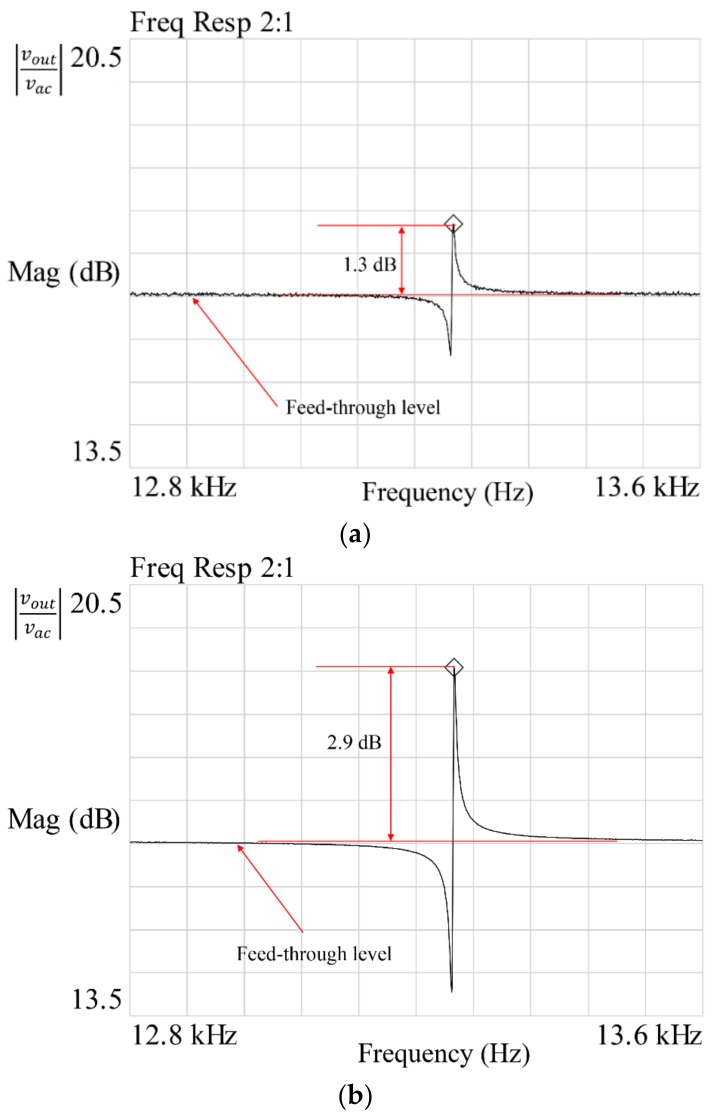
Frequency response function: (**a**) condition 1 and (**b**) condition 2.

It is clear from the experimental results that the resonant frequency measurement suffered from systematic errors only. These results agree with the work by Lee [18]. However, quality factor measurement suffers from greater random errors with a small driving force. The results indicate that *Q* factor measurement depends directly on the applied force. The random errors were reduced by increasing the electrostatic force. In this paper, we focus on the electrostatic force. The influence of the applied AC and DC voltages was considered, however, environmental factors, such as temperature and pressure, also affect the measurement setup, and can result in measurement variations. The typical FRF of the resonator with conditions 1 and 2 is shown in Figure 9.

## 4. Conclusions and Future Work

In this paper, the measurement uncertainties in capacitive MEMS resonators were studied. The influence of the electrostatic driving force on the resonant characteristics was demonstrated. We found that increasing the driving force results in lower measurement uncertainty. The resonant frequency is influenced by systematic errors, and is independent of the applied driving force, but the quality factor measurement directly depends on the applied driving force. For a small driving force, the random noise is quite high, which results in greater measurement variations.

The effects of AC and/or DC voltages were studied independently. By increasing the DC voltage, the transmission magnitude of the sense signal was increased. However, increasing the AC voltage results in a high feed-through noise level, while the transmission magnitude remains the same. It was observed that the measurement variations were reduced by increasing both the AC and DC voltages, regardless of parasitic feed-through level. 

This study was performed on a high Q resonator. The resonant characteristics were directly measured by the signal analyzer, which works quite well in case of high Q due to sharp peaks. However, for extremely low Q resonators, the performance and accuracy of the algorithm used by the signal analyzer may be degraded. In addition, the device used in this study was actuated differentially, using comb drive, for which the effect of non-linearity was neglected. In our future work, we will try to quantify the effect of non-linearity on the measurement variations in resonant characteristics of MEMS resonators.

## Figures and Tables

**Table 1:** Test conditions for the signal analyzer.

Test	Measured Parameter	Window Type	Average	Frequency Span	Frequency Resolution
Frequency domain analysis	Frequency response, Q factor	Uniform	Type: RMS, number 5	800 Hz	800 lines/Hz

**Table 2:** Electrostatic force conditions.

Parameters	Condition 1	Condition 2
Number of devices	10	10
Number of trials	3	3
*V_AC_*	200 mV_pk_	1 V_pk_
*V_DC_*	2 V	5 V
Operators	2	2

**Table 3:** Condition 1 Gage R&R for resonant frequency $\left(f_{r}\right).$

Source	VarComp	% Contribution (of VarComp)	stDev (SD)	Study Var (6 × SD)	% Study Var (% SV)
Total Gage R&R	0.8	0.0	1.0	5.3	0.4
Repeatability	0.8	0.0	1.0	5.3	0.4
Reproducibility	0.0	0.0	0.0	0.0	0.0
Operators	0.0	0.0	0.0	0.0	0.0
Part-to-part	42,362.7	100.00	205.8	1234.9	100.0
Total variation	42,363.4	100.00	205.8	1234.9	100.0
Number of distinct categories	329.0				

**Table 4:** Condition 1 Gage R&R for the Q factor.

Source	VarComp	% Contribution (of VarComp)	stDev (SD)	Study Var (6 × SD)	% Study Var (% SV)
Total Gage R&R	40,809.0	9.6	202.0	1212.0	30.9
Repeatability	40,809.0	9.6	202.0	1212.0	30.9
Reproducibility	0.0	0.0	0.0	0.0	0.0
Operators	0.0	0.0	0.0	0.0	0.0
Part-to-part	386,300.0	90.5	621.5	3729.1	95.1
Total variation	427,110.0	100.0	652.5	3921.2	100.0
Number of distinct categories	4.0				

**Table 5:** Condition 2 Gage R&R for the resonant frequency $\left(f_{r}\right).$

Source	VarComp	% Contribution (of VarComp)	stDev (SD)	Study Var (6 × SD)	% Study Var (% SV)
Total Gage R&R	0.1	0.0	0.2	1.5	0.1
Repeatability	0.1	0.0	0.2	1.5	0.1
Reproducibility	0.0	0.0	0.0	0.1	0.0
Operators	0.0	0.0	0.0	0.1	0.0
Part-to-part	42,392.9	100.0	205.9	1235.4	100.0
Total variation	42,392.9	100.0	205.9	1235.4	100.0
Number of distinct categories	1184.0				

**Table 6:** Condition 2 Gage R&R for the Q factor.

Source	VarComp	% Contribution (of VarComp)	stDev (SD)	Study Var (6 × SD)	% Study Var (% SV)
Total Gage R&R	279.0	0.1	16.7	100.1	2.5
Repeatability	279.0	0.1	16.7	100.1	2.5
Reproducibility	0.0	0.0	0.0	0.0	0.0
Operators	0.0	0.0	0.0	0.0	0.0
Part-to-part	432,933.0	99.9	658.0	3947.9	99.9
Total variation	433,212.0	100.0	658.2	3949.1	100.0
Number of distinct categories	55.0				
